# Safety Evaluation of Tetanus, Diphtheria, and Acellular Pertussis Vaccine (Tdap) During Pregnancy Among Vietnamese Women

**DOI:** 10.3390/vaccines13101036

**Published:** 2025-10-08

**Authors:** Hien Minh Nguyen, Nhat Thang Tran, Quoc Huy Pham, Huu Nghia Cao

**Affiliations:** 1 Department of Physiology–Pathophysiology–Immunology, Medical School, University of Medicine and Pharmacy, Ho Chi Minh City 700000, Vietnam; 2 Department of Obstetrics and Gynecology, University Medical Center, Ho Chi Minh City 700000, Vietnam; thang.tn@umc.edu.vn; 3 Pasteur Institute, Ho Chi Minh City 700000, Vietnam; huypq@pasteurhcm.edu.vn (Q.H.P.); nghiach@pasteurhcm.edu.vn (H.N.C.)

**Keywords:** Tdap, pregnant women, local adverse events, systemic adverse events, vaccine safety

## Abstract

*Background:* In Vietnam, the Ministry of Health recently approved the use of Tdap vaccines—Boostrix (2022) and Adacel (2024)—for administration during pregnancy, aiming to provide passive antibody transfer to protect newborns against pertussis and tetanus from birth. However, uptake remains low, largely because Tdap is not included in the National Expanded Program on Immunization, vaccine hesitancy persists among obstetricians, and local safety data in pregnancy are limited. *Methods:* We conducted a prospective cohort study from September 2023 to September 2024 involving 485 pregnant women between 27 and 36 weeks of gestation at two major hospitals in Ho Chi Minh City: University Medical Center and Gia Dinh People’s Hospital. Participants received either Tdap or monovalent tetanus toxoid vaccine (TT) as a comparator. *Results:* Among women in the Tdap group, 49.8% reported at least one adverse event following immunization (AEFI). Local reactions were the most frequent, primarily injection-site pain (43.8%). Fatigue (12.8%) was the most common systemic reaction, followed by headache (3.9%). Grade 3 AEFIs occurred in 5% of the Tdap group and included extensive local reactions (erythema or swelling > 3 cm), high-grade fever (≥40 °C), and severe fatigue interfering with daily activities or requiring hospitalization. Women receiving Tdap had 1.52-fold higher injection-site pain compared with those receiving TT (95% CI: 0.060–0.782). Importantly, co-administration of Tdap with inactivated quadrivalent influenza vaccine (IIV4) did not increase the risk of AEFIs. Furthermore, no evidence was found that Tdap vaccination adversely affected the course of pre-existing maternal comorbidities, which remained stable throughout pregnancy. *Conclusions:* This first large-scale Vietnamese cohort provides reassuring evidence on the safety of Tdap vaccination during pregnancy. These findings support broader implementation of maternal Tdap immunization, including concomitant administration with IIV4, to protect both mothers and infants.

## 1. Introduction

Pertussis, commonly known as whooping cough, has re-emerged as a significant global public health challenge. According to data from the World Health Organization (WHO), there were 158,910 reported cases of pertussis globally [1] in 2023. While the number of cases has fluctuated over the years, the recent widespread outbreaks underscore the continued threat posed by the disease. The WHO’s 2018 data indicates that pertussis was responsible for approximately 161,000 deaths worldwide [1], with the majority occurring in children under five years of age. Of particular concern is the rise in pertussis cases among infants under 3 months of age, a population especially vulnerable due to incomplete immunization [2].

In Vietnam, pertussis vaccination coverage among children under 1 year of age has been consistently maintained at over 85% for many years [3,4]. As a result, the incidence of pertussis in children declined from 84.4 per 100,000 population in 1984 to only 0.46 per 100,000 in 2004 [4]. However, during the past five years (2015–2020), this rate has shown a slight upward trend, reaching 0.7–1.06 per 100,000 population [4]. The COVID-19 pandemic resulted in vaccine shortages in Vietnam from 2021 to 2023, during which the DTP3 coverage rate fell to 65% [3]. Among hospitalized pertussis cases under 5 years old, about 64% occurred in infants younger than three months [5]. Since 2020, the Vietnam Association of Preventive Medicine has recommended the administration of one dose of reduced-dose tetanus, diphtheria, and acellular pertussis vaccine (Tdap) for pregnant women between 27 and 36 weeks of gestation during each pregnancy. In 2021, the Vietnam Association of Obstetrics and Gynecology issued a similar recommendation, further emphasizing both the role and legal basis for efforts to incorporate Tdap vaccination into the national immunization program.

In Vietnam, evidence on the immunogenicity of the Tdap vaccine remains limited [6,7], and no large-scale safety studies in pregnant women have been reported to date. In line with updated global recommendations for maternal Tdap immunization and the national context, a prospective study was conducted at the University Medical Center Ho Chi Minh City and Gia Dinh People’s Hospital between September 2023 and September 2024. The primary objective was to evaluate the safety profile of Tdap vaccination during pregnancy. Specifically, the study aimed to (1) assess the clinical course of underlying maternal conditions before and after Tdap administration and (2) examine local and systemic adverse events following vaccination. We hypothesized that Tdap vaccination during pregnancy is safe and shows no difference in safety compared with tetanus toxoid (TT), which has been used in Vietnamese pregnant women for over 30 years and remains well tolerated when co-administered with the influenza vaccine.

## 2. Materials and Methods

### 2.1. Study Design

A prospective cohort study was conducted.

### 2.2. Study Population

Participants were pregnant women residing in Ho Chi Minh City who received the reduced-dose tetanus, diphtheria, and acellular pertussis vaccine (Tdap; Boostrix^®^, GlaxoSmithKline, Rixensart, Belgium). The comparator vaccine was monovalent tetanus toxoid (TT; adsorbed tetanus vaccine, Institute of Vaccines and Medical Biologicals IVAC, Nha Trang, Vietnam). For concomitant vaccination, the inactivated quadrivalent influenza vaccine (Influvac Tetra^®^, Abbott, Weesp, The Netherlands) was administered together with Tdap.

### 2.3. Study Setting and Duration

The study took place at the Vaccination Unit of the University Medical Center Ho Chi Minh City and the Obstetrics Department of Gia Dinh People’s Hospital from September 2023 to September 2024.

### 2.4. Inclusion Criteria

Eligible participants were pregnant women aged 18 years or older who provided written informed consent to participate in the study.

### 2.5. Exclusion Criteria

Participants were excluded if they failed to complete the questionnaire, had a known hypersensitivity to vaccine components, or had a history of severe adverse events following vaccination with diphtheria-, pertussis-, or tetanus-containing vaccines.

### 2.6. Sample Size

The required sample size was calculated using the formula for estimating a proportion:

$$N\geq \frac{Z_{1-a/2}^{2}\;p(1-p)\;}{d^{2}}$$

The sample size (N) was estimated using the single-proportion formula, with *Z* = 1.96 (95% confidence), *d* = 0.05, and *α* = 0.05. Based on *p* = 23.9% from the study by Vera Lúcia Gattás [8], the minimum required sample size was N ≥ 280.

### 2.7. Statistical Analysis

Data entry was performed using Microsoft Excel 365, and statistical analyses were conducted with JASP software version 0.18.0.0 (JASP Team, Amsterdam, The Netherlands). A significance level of 0.05 was applied for all analyses.

### 2.8. Ethical Considerations

The study protocol was reviewed and approved by the Institutional Review Board of the University of Medicine and Pharmacy at Ho Chi Minh City (approval No. 579/HĐĐĐ-ĐHYD, dated 22 March 2023) and the Ethics Committee of Gia Dinh People’s Hospital (approval No. 92/NDGĐ-HĐĐĐ, dated 10 August 2023).

### 2.9. Data Collection Methods

All eligible pregnant women completed a structured questionnaire and were randomly assigned to either the tetanus toxoid (TT) or Tdap group. Participant lists were verified against hospital records for accurate age selection. Enrollment was conducted after informed consent.

Data were collected from vaccination through delivery and until infants reached two months of age. Adverse events following immunization (AEFI) were monitored using passive methods (telephone calls, email questionnaires) and active methods (home or hospital visits). Infant outcomes were documented from delivery and vaccination records.

Outcomes were classified into two categories: local reactions (pain, redness, swelling/induration) and systemic reactions (fatigue, fever, anorexia, nausea/vomiting, rash, dizziness, headache). Data were obtained through self-report, direct observation, and clinical records. Redness and swelling were measured by diameter, pain was assessed by its impact on movement, and systemic events were evaluated by symptom frequency, temperature measurement, or interference with daily activities.

All adverse events were graded on a 0–3 severity scale: Grade 0 = absent; Grade 1 = mild, no impact on daily activity; Grade 2 = moderate, some limitation; Grade 3 = severe, marked limitation requiring medical care or hospitalization.

## 3. Results

### 3.1. Demographic Characteristics of Pregnant Participants

**Figure 1:**
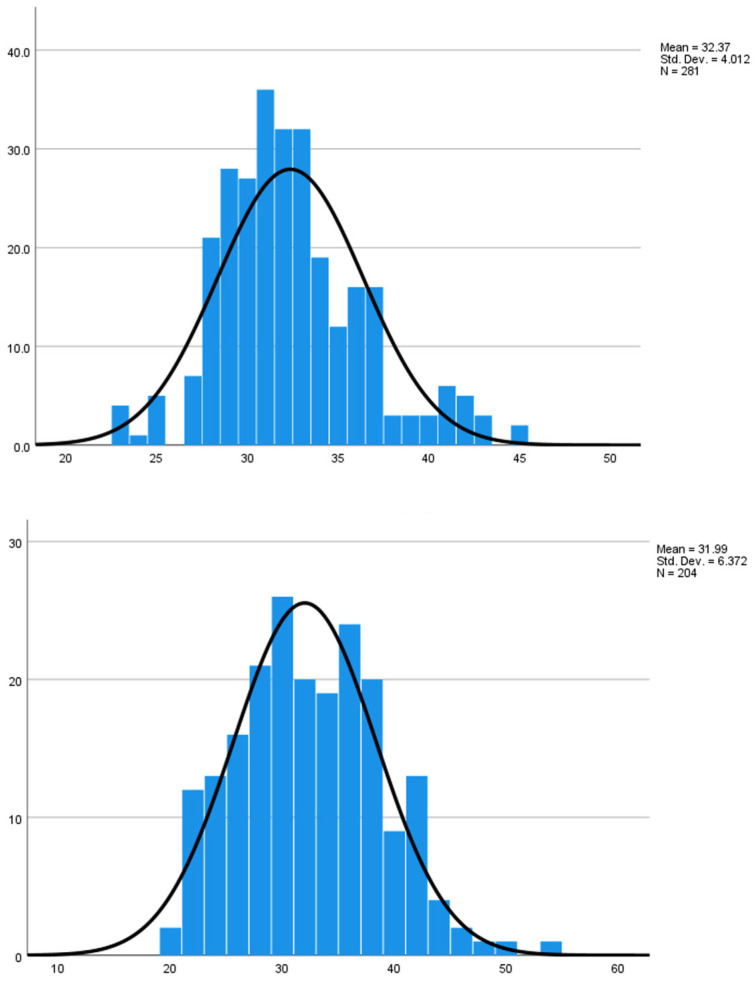
Age distribution of pregnant participants in the Tdap and TT groups.

The upper panel shows the age distribution of pregnant women receiving Tdap (n = 281; mean age = 32.37 years). The lower panel shows the age distribution of pregnant women receiving tetanus toxoid (TT) (n = 204; mean age = 31.99 years), which is comparable to the Tdap group (Table 1 and Figure 1).

### 3.2. Maternal Comorbidities Before and During Pregnancy

#### 3.2.1. Pre-Existing Conditions

**Figure 2:**
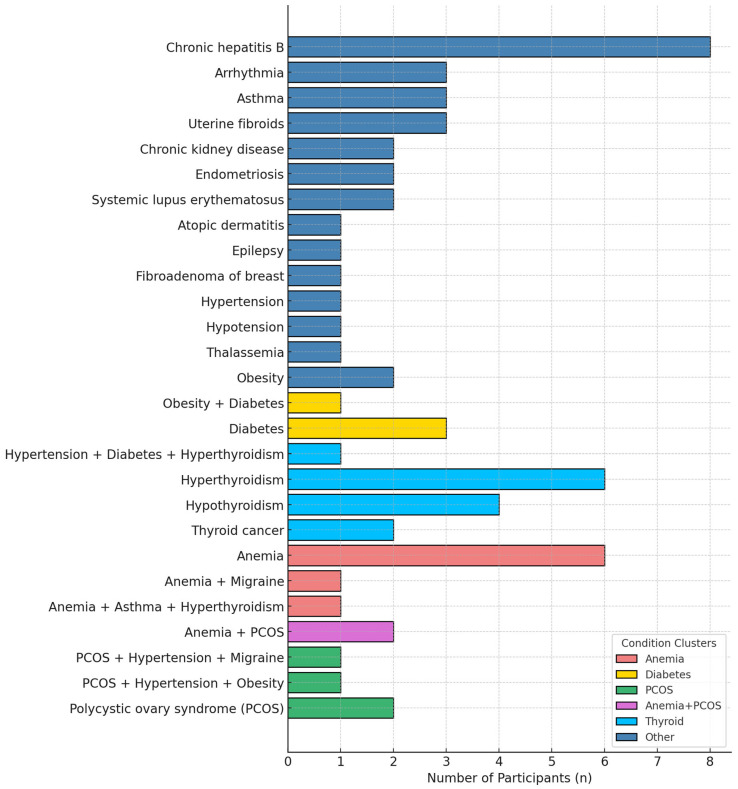
Pre-existing medical conditions among pregnant women receiving Tdap vaccination (n= 64).

At enrollment, 64 of 281 pregnant women (22.8%) in the Tdap group presented with underlying comorbidities. Among these 64 women, the most prevalent conditions were thyroid disorders (21.9%; including hyperthyroidism, hypothyroidism, and thyroid cancer), anemia (15.6%), and chronic hepatitis B (12.5%). Diabetes accounted for 6.3%, while polycystic ovary syndrome (PCOS), obesity, and hypertension each accounted for 4.7%. All pre-existing conditions remained clinically stable throughout pregnancy, with no evidence of worsening following vaccination (Figure 2).

#### 3.2.2. Pregnancy-Related Conditions

**Figure 3:**
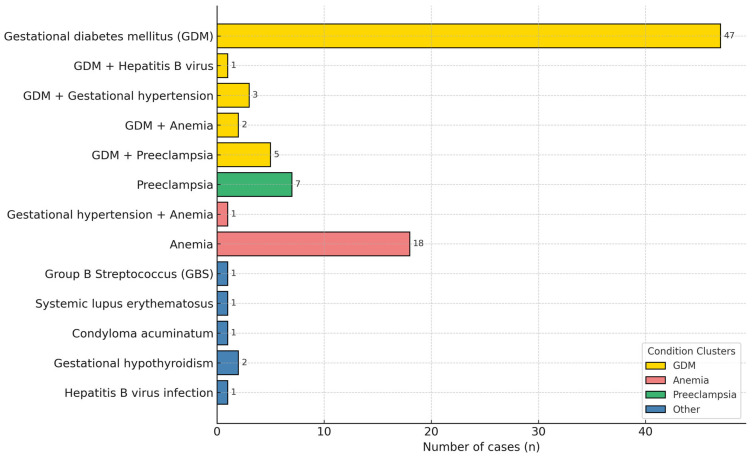
Pregnancy-related conditions among pregnant women receiving Tdap vaccination (n = 90).

Of the 281 pregnant women who received Tdap, 90 (32.0%) were diagnosed with pregnancy-related conditions, 80% of which had been identified before Tdap administration. Among these 90 women, gestational diabetes mellitus (GDM) was the most prevalent (64.4%), followed by anemia (23.3%) and preeclampsia (13.3%). These percentages reflect the overall prevalence of each condition, including both isolated and coexisting cases. No severe progression or adverse fetal outcomes were observed in association with vaccination (Figure 3).

### 3.3. Description of Adverse Event Following Immunization (AEFI) Following Tdap Vaccination

Among 281 pregnant women who received the Tdap vaccine, local adverse events (AEs) were the most frequently reported, with pain at the injection site observed in 43.8% of participants, followed by erythema (3.9%) and swelling (3.9%) (Table 2). The majority of pain cases were graded as mild (Grade 1, 23.5%) or moderate (Grade 2, 17.8%), while only a small proportion were severe (Grade 3, 2.5%).

Regarding systemic AEs, the most common reactions were fatigue (12.8%) and headache (3.9%), while fever (1.1%), dizziness (1.1%), and loss of appetite (2.1%) were infrequent (Table 3). All reported systemic events were self-limited and resolved without medical treatment.

A comparative analysis between women receiving Tdap and those receiving tetanus toxoid (TT) (n = 485) showed that the overall distribution of local and systemic AEs was similar between the two groups (Table 4). However, injection-site pain occurred significantly more often in the Tdap group compared to the TT group (43.8% vs. 26.8%, *p* = 0.008). No significant differences were observed for erythema, swelling, or systemic events such as fatigue, fever, dizziness, or headache (all *p* > 0.05).

Statistical analysis indicated that pregnant women who received Tdap had more than five-fold higher odds of experiencing grade 3 adverse events compared with those who received tetanus toxoid (TT) (OR = 5.30, 95% CI: 1.19–23.56, *p* = 0.018). However, this odds ratio represents relative odds rather than absolute risk, and the absolute number of grade 3 events was low in both groups, indicating that the clinical significance of this finding is likely limited. Importantly, this difference was mainly attributable to mild local pain at the injection site, with no serious or unexpected events reported (Table 5).

AEFIs were generally more frequent in the Tdap + IIV4 group compared with Tdap alone, particularly for local reactions such as pain, erythema, swelling, and headache. However, no significant differences in the frequency of local or systemic adverse events were observed between women who received Tdap alone and those who received concomitant Tdap plus inactivated quadrivalent influenza vaccine (IIV4) (Table 6).

## 4. Discussion

In Vietnam, maternal immunization has for decades relied almost exclusively on tetanus toxoid (TT), widely administered in maternity care settings [9]. This long-standing practice has shaped both provider habits and maternal expectations, making the shift to Tdap far from straightforward. A knowledge, attitudes, and practices (KAP) survey at University Medical Center Ho Chi Minh City (UMC) showed that provider hesitation and maternal misconceptions remain major obstacles. While 71% of pregnant women expressed willingness to receive Tdap, over half held inaccurate beliefs about its potential harm to the mother or fetus [10]. Although Tdap was officially licensed for use in pregnancy in 2022, it has not yet been included in the national Expanded Program on Immunization (EPI). As a result, it is only available as a self-paid service, whereas TT remains the sole vaccine mandated for pregnant women under current regulations (Vietnam Ministry of Health Circular No. 10, 2024) [3,5,11]. In addition, many obstetricians remain hesitant to recommend Tdap over TT, citing concerns that such changes may be difficult to communicate to pregnant women, who might perceive deviations from traditional practice as potential risks to maternal and infant health. Similar barriers in the United States—limited access, provider hesitancy, and maternal safety concerns—were reported when Tdap was first introduced during pregnancy [10,12].

The first Tdap doses administered to pregnant women in Vietnam were conducted at the UMC during the COVID-19 pandemic [13]. Tdap vaccination has demonstrated a favorable safety profile within 7 days post-administration in pregnant women between 27 and 36 weeks of gestation, even when co-administered with the COVID-19 vaccine or the influenza vaccine (IIV4). In our previous small-scale study conducted at University Medical Center Ho Chi Minh City in 2021, which evaluated the co-administration of Tdap and COVID-19 vaccines in 78 pregnant women, adverse events following immunization (AEFIs) were mostly mild and self-limiting, and no serious vaccine-related events were reported [13]. Among systemic AEs, fatigue was the most frequently reported (28.2%), followed by fever (10.3%). Local AEs primarily included pain at the injection site (66.7%), with redness and/or swelling observed in 14.1% of participants and itching in 9%. These results are consistent with our subsequent observational data on concomitant Tdap and IIV4 administration, which also showed predominantly mild and self-limited AEs (Table 6). As the safety of maternal immunization is a primary concern, it is also important to address the safety of co-administration with other vaccines to build confidence and acceptance in vaccination programs. This information is essential to inform and support the development of future maternal influenza immunization policies in Vietnam. Our findings on Tdap vaccination (Table 2 and 3) are broadly consistent with studies conducted in Asian pregnant populations, which also reported predominantly mild AEFIs and no new safety concerns [14,15,16]. Our prospective cohort adds important evidence that Tdap is safe for Vietnamese pregnant women, showing an adverse event profile comparable to tetanus toxoid (Table 4). Building this local safety evidence is a crucial first step toward gaining the trust of both pregnant women and obstetricians, which is essential for broader acceptance in Vietnam. Once this confidence is established, national policy updates to replace TT with Tdap can be implemented more effectively to better protect both mothers and their infants.

In addition, most Vietnamese pregnant women have little or no prior experience with influenza vaccination, as influenza immunization has not been routinely recommended during pregnancy. This unfamiliarity can reinforce hesitancy when Tdap and influenza vaccines are offered together. However, our study demonstrated that co-administration of Tdap and inactivated quadrivalent influenza vaccine (IIV4) was safe, with no increase in adverse events compared with Tdap alone. This reassurance is consistent with findings from other high-income country studies [17,18]. Together, these data align with World Health Organization recommendations endorsing both Tdap and influenza vaccines during pregnancy [19,20] to enhance maternal and neonatal protection.

## 5. Conclusions

This study shows that Tdap vaccination is safe for Vietnamese pregnant women, including those with comorbidities or pregnancy-related conditions, with an adverse event profile comparable to tetanus toxoid (TT). Co-administration with the influenza vaccine (IIV4) was also well tolerated. These findings support our hypothesis and provide essential national evidence to guide future maternal immunization policies in Vietnam, including the integration of co-administration strategies into the Expanded Program on Immunization (EPI).

## Figures and Tables

**Table 1:** Baseline characteristics of pregnant participants (N = 485).

Characteristics	Tdap (n = 281)	TT (n = 204)
Number of pregnancies		
1	164 (58.36%)	90 (44.12%)
2	103 (36.66%)	79 (38.72%)
3	14 (4.98%)	34 (16.67%)
≥4	0 (0%)	1 (0.49%)
Number of fetuses		
Singleton	279 (99.29%)	204 (100%)
Twin	2 (0.71%)	0 (0%)
Multiple (>2)	0 (0%)	0 (0%)
Maternal age, years (mean ± SD)	32.37 ± 4.01	31.99 ± 6.37

**Table 2:** Local adverse events (AE) associated with Tdap vaccination during pregnancy (n = 281).

Type of Local AE	Reaction	Severity Grade	Number of Cases	Proportion (%)
Pain *	No	0	158	56.23
	Yes (43.77%)	1	66	23.49
		2	50	17.79
		3	7	2.49
Erythema	No	0	270	96.09
	Yes (3.91%)	1	9	3.20
		2	2	0.71
Swelling	No	0	270	96.09
	Yes (3.91%)	1	8	2.85
		2	3	1.07

* Nearly half of the pregnant women (43.77%) experienced injection-site pain of varying severity, with mild pain (Grade 1) being the most common. Two pregnant women reported arm pain extending from the injection site to the whole arm after Tdap vaccination. Within 1–2 h post-vaccination, the pain radiated down to the little finger, was most intense at the injection site, diminished gradually toward distal regions, and resolved completely within 3 days.

**Table 3:** Systemic adverse events associated with Tdap vaccination during pregnancy (n = 281).

Type of systemic AE	Reaction	Severity Grade	Number of Cases	Proportion (%)
Fatigue	No	0	245	87.19
	Yes (12.81%)	1	26	9.25
		2	7	2.49
		3	3	1.07
Fever	No	0	278	98.93
	Yes (1.07%)	1	2	0.71
		2	1	0.36
Loss of appetite	No	0	275	97.86
	Yes (2.14%)	1	4	1.42
		2	1	0.36
		3	1	0.36
Nausea	No	0	281	100.00
Rash	No	0	281	100.00
Dizziness	No	0	278	98.93
	Yes (1.07%)	1	1	0.36
		2	1	0.36
		3	1	0.36
Headache	No	0	270	96.09
	Yes (3.91%)	1	6	2.14
		2	3	1.07
		3	2	0.71

**Table 4:** Local and systemic adverse events (AEs) among pregnant women receiving Tdap versus TT (N = 485).

Type of AEs	Response	Severity	Tdap (%)	TT (%)	*p*-Value
Local AEs					
Pain	No	0	158 (32.6%)	130 (26.8%)	0.008 **
	Yes	1	66 (13.6%)	54 (11.1%)	
		2	50 (10.3%)	20 (4.1%)	
		3	7 (1.4%)	0 (0%)	
Erythema	No	0	270 (55.7%)	196 (40.4%)	0.443
	Yes	1	9 (1.9%)	8 (1.6%)	
		2	2 (0.4%)	0 (0%)	
Swelling	No	0	270 (55.7%)	192 (39.6%)	0.598
	Yes	1	8 (1.6%)	17 (1.9%)	
		2	3 (0.6%)	3 (0.6%)	
Systemic AEs					
Fatigue	No	0	245 (50.5%)	186 (38.4%)	0.551
	Yes	1	26 (5.4%)	14 (2.9%)	
		2	7 (1.4%)	3 (0.6%)	
		3	3 (0.6%)	1 (0.2%)	
Fever	No	0	278 (57.3%)	203 (41.9%)	0.661
	Yes	1	2 (0.4%)	1 (0.2%)	
		2	1 (0.2%)	0 (0%)	
Loss of appetite	No	0	275 (56.7%)	201 (41.4%)	0.692
	Yes	1	4 (0.8%)	3 (0.6%)	
		2	1 (0.2%)	0 (0%)	
		3	1 (0.2%)	0 (0%)	
Nausea	No	0	281 (57.9%)	203 (41.9%)	0.240
	Yes	1	0 (0%)	1 (0.2%)	
Rash	No	0	281 (57.9%)	204 (42.1%)	–
	Yes	1	0 (0%)	0 (0%)	
Dizziness	No	0	278 (57.3%)	202 (41.6%)	0.532
	Yes	1	1 (0.2%)	2 (0.4%)	
		2	1 (0.2%)	0 (0%)	
		3	1 (0.2%)	0 (0%)	
Headache	No	0	270 (55.7%)	201 (41.4%)	0.148
	Yes	1	2 (0.4%)	2 (0.4%)	
		2	7 (1.4%)	0 (0%)	
		3	2 (0.4%)	1 (0.2%)	

** Injection-site pain: Among pregnant women, a statistically significant difference was observed between the two vaccine groups (*p* = 0.008). Ordinal regression analysis confirmed an association between vaccine type and pain severity (*p* = 0.022), with women receiving Tdap having 1.52 times higher odds of reporting greater injection-site pain compared with those receiving TT (95% CI: 1.062–2.186).

**Table 5:** Comparison of total grade 3 adverse events (AEs) between vaccine groups.

AE type	Reaction	Tdap (n = 281)	TT (n = 204)	*p*-Value	OR (95% CI)
Total grade 3 AEs	No	267 (95.0%)	202 (99.0%)	0.018	5.30 (1.19–23.56) *p*-value calculated using Fisher’s exact test
	Yes	14 (5.0%)	2 (1.0%)		

**Table 6:** Comparison of local and systemic adverse events between Tdap alone and concomitant Tdap plus inactivated quadrivalent influenza vaccine (IIV4).

Adverse Event	Tdap (n = 204)	Tdap + IIV4 (n = 77)	*p*-Value	OR (95% CI)
**Local AEs**				
Pain	87 (42.6%)	37 (48.1%)	0.416	1.244 (0.735–2.105)
Erythema	6 (2.9%)	5 (6.5%)	0.171	2.292 (0.679–7.740)
Swelling	6 (2.9%)	5 (6.5%)	0.171	2.292 (0.679–7.740)
**Systemic AEs**				
Fatigue	26 (12.7%)	10 (13.0%)	0.957	1.022 (0.468–2.233)
Fever	2 (1.0%)	1 (1.3%)	0.817	1.329 (0.119–14.869)
Loss of appetite	5 (2.5%)	1 (1.3%)	0.551	0.524 (0.060–4.556)
Dizziness	2 (1.0%)	1 (1.3%)	0.817	1.329 (0.119–14.869)
Headache	6 (2.9%)	5 (6.5%)	0.171	2.292 (0.679–7.740)
Nausea	0 (0%)	0 (0%)	—	—
Rash	0 (0%)	0 (0%)	—	—

## Data Availability

The data presented in this study are available on request from the corresponding author. The data are not publicly available due to privacy and ethical restrictions.
